# The role of apoptotic cell death in the radiosensitising effect of gemcitabine

**DOI:** 10.1038/sj.bjc.6605145

**Published:** 2009-08-11

**Authors:** B Pauwels, J B Vermorken, A Wouters, J Ides, S Van Laere, H A J Lambrechts, G G O Pattyn, K Vermeulen, P Meijnders, F Lardon

**Affiliations:** 1 Laboratory of Cancer Research and Clinical Oncology, University of Antwerp, Universiteitsplein 1, 2610 Wilrijk, Belgium; 2 Translational Cancer Research Group, Laboratory of Pathology, University of Antwerp, Universiteitsplein 1, 2610 Wilrijk, Belgium; 3 Laboratory of Experimental Hematology, University of Antwerp, Wilrijkstraat 10, 2650 Edegem, Belgium; 4 University Radiotherapy, ZNA Middelheim, Lindendreef 1, 2020 Antwerp, Belgium

**Keywords:** gemcitabine, radiation, radiosensitisation, cell cycle, apoptosis

## Abstract

**Background:**

The aim of this study was to evaluate the radiosensitising effect of gemcitabine, in terms of cell-cycle progression, induction of apoptosis, and to investigate the molecular events regulating apoptosis.

**Methods:**

Tumour cells were treated with gemcitabine, radiation, or the combination. 0–72 h after treatment, cells were collected for cell-cycle analysis and apoptosis determination. Caspase 8 and 9, Bid and tBid expression were determined by western blot. The mitochondrial membrane potential was determined using flow cytometry. An RT^2^ *Profiler* PCR Array for human apoptotic genes was performed after the combination or TRAIL treatment.

**Results:**

Gemcitabine and radiation resulted in an early S-phase block immediately after treatment, after which the cells moved synchronously through the cell cycle. When cell-cycle distribution returned to pre-treatment levels, an increased induction of apoptosis was observed with activation of caspase 8 and 9 and a reduction of the mitochondrial membrane potential. Gene expression after treatment with radiosensitising conditions was comparable with expression after the TRAIL treatment.

**Conclusion:**

A role for the cell-cycle perturbations and the induction of apoptosis could be attributed to the radiosensitising effect of gemcitabine. Apoptosis induction was comparable with the apoptotic pathway observed after the TRAIL treatment, that is the involvement of the extrinsic apoptosis pathway.

Killing of tumour cells by cytotoxic therapies, such as chemotherapy and *γ*-irradiation, is in some tumours predominantly mediated by triggering apoptosis (Brown and Wouters, 2001; Debatin and Krammer, 2004). This might occur either as a primary event induced by therapy or as a secondary event after lethal damage to the cell (Tannock and Lee, 2001). A family of cystein-dependent aspartate directed proteases, called caspases, is responsible for the proteolytic cleavage of cellular proteins leading to the characteristic apoptotic features. Currently, two pathways for activating caspases have been studied in detail, that is the mitochondrial ‘intrinsic’ pathway and the transmembrane ‘extrinsic’ pathway. Both pathways share the same effector caspases (caspase 3, 6, and 7).

The intrinsic pathway is under control of the Bcl-2 protein family. Permeabilisation of the outer mitochondrial membrane induces the leakage of proapoptotic molecules from the mitochondrial intermembrane space. In the cytosol, cytochrome *c* induces the oligomerisation of apoptosis protease activating factor 1 (Apaf-1) in the presence of ATP or dATP. Apoptosis protease activating factor 1 oligomers recruit procaspase 9 molecules in a complex called the ‘apoptosome’. The release of mature caspase 9 activates additional caspase 9 molecules as well as caspase 3 and 7. In turn, caspase 3 activates downstream caspase cascades. At the same time, the release of Smac/DIABLO and HtrA2/Omi neutralises the inhibitory effects of inhibitors of apoptosis proteins on caspase 3, 7, and 9.

Plasma membrane receptors for triggering external apoptosis signalling belong to the tumour necrosis factor (TNF)-receptor superfamily. The best-studied death receptor is Fas; binding of Fas ligand (FasL) leads to receptor trimerisation and recruitment of specific adaptor proteins. The Fas receptor contains a death domain (DD) in its cytoplasmatic region, which interacts with the adaptor protein, Fas-associating DD protein (FADD), forming a death receptor-induced signalling complex (DISC). Besides a DD, FADD contains a death effector domain (DED) and this recruits the DED-containing procaspase 8 into the DISC. Procaspase 8 will be proteolytically activated to the enzymatically active caspase 8, which in turn will activate downstream effector caspases. Other death receptors activate caspases in a similar manner. Depending on the cell type, activated caspase 8 induces apoptosis by two different signalling pathways. In type I cells, large amounts of active caspase 8 formed at the DISC induce direct activation of procaspase 3 independently of mitochondria. In type II cells, the presence of only very little DISC and small amounts of caspase 8 is insufficient to activate procaspase 3 directly and therefore amplification of the apoptotic signal through the mitochondrial apoptosis pathway is required. Instead, caspase 8 cleaves the ‘BH3-only protein’ Bid, generating an active fragment (tBid) that activates the mitochondrial death pathway (Green, 2000; Debatin and Krammer, 2004; Fulda and Debatin, 2006).

The nucleoside analogue gemcitabine (dFdC) has shown promising clinical effectiveness against a range of solid tumours. The cytotoxic effect of this agent is mediated by the induction of apoptotic cell death as shown by Huang and Plunkett (1995a). In addition, gemcitabine has shown both in laboratory and clinical studies to be a potent radiosensitiser (Pauwels *et al*, 2005a), but the exact mechanism of radiosensitisation remains as yet unknown. Interesting in that respect is the fact that gemcitabine-induced accumulation of cells in the S phase appears to be required for maximal radiosensitisation (Pauwels *et al*, 2005a; Shewach and Lawrence, 2007). Taking into account that the S phase is not reported to be the most radiosensitive phase of the cell cycle (Sinclair, 1972; Tang *et al*, 1997), the question remains whether the influence on the cell cycle indeed plays a role in radiosensitisation. Studies investigating the radiosensitising effect of gemcitabine hypothesised that the ability of cells to progress through the S phase after gemcitabine and radiation may be a key for radiosensitisation to occur (Ostruszka and Shewach, 2000; Mose *et al*, 2003). It may suggest that cells progressing beyond the S-phase block might accumulate proapoptotic signals, caused by both radiation and gemcitabine, resulting in increased cell death. It has also been hypothesised that radiosensitisation by gemcitabine is the result of lowering the threshold for radiation-induced apoptosis (Doyle *et al*, 2001).

Therefore, the aims of this study were to evaluate the cell-cycle progression and to study the induction of apoptosis, in two types of human tumour cells, after treatment with gemcitabine and/or radiotherapy. In addition, the molecular events that regulate apoptosis were explored. Understanding these events may provide important new opportunities for pathway-based rational therapy and for drug development.

## Materials and methods

### Cell lines

The cell lines used in this study were ECV304 (mutant (mt) p53), a human epidermoid bladder cancer cell line and H292 (wild-type (wt) p53), a human mucoepidermoid lung cancer cell line. H292 was cultured in RPMI-1640 medium, supplemented with L-glutamine, sodium pyruvate, and 10% fetal calf serum (Invitrogen, Merelbeke, Belgium). ECV304 was cultured in medium-199 supplemented with 10% fetal calf serum. Cultures were maintained in exponential growth in a humidified atmosphere at 37°C under 5% CO_2_/95% air. For subsequent experiments, cells were collected by trypsinisation, counted, and plated as specified below.

As we reported earlier, the IC_50_ value of 24 h treatment with gemcitabine was 3.05±0.49 for ECV304 cells and 7.99±0.77 for H292 cells (Pauwels *et al*, 2003a).

### Cell survival after treatment with gemcitabine and radiation

Cells were plated in 48-well plates and treated as we described earlier (Pauwels *et al*, 2003a, 2003c, 2005b). Gemcitabine was added during 24 h, immediately followed by radiation or immediately after radiation (room temperature, 0–8 Gy, linear accelerator). After 7 days, the survival was determined by the sulforhodamine B (SRB) assay as described earlier (Pauwels *et al*, 2003a, 2003b). This method was comparable with the clonogenic assay taking in account some critical aspects (Pauwels *et al*, 2003b).

### Cell cycle effect of gemcitabine and/or radiation

Cells were plated in 6-well plates as described earlier (Pauwels *et al*, 2003c). To ensure exponential growth during the experiments, seeding densities ranged from 0.3 to 0.5 × 10^5^ cells per well depending of culture time after treatment. Cells were treated with 8 nM of gemcitabine for 24 h, or irradiated (4 Gy), or treated with 8 nM gemcitabine for 24 h immediately before 4 Gy radiation. Medium was replaced immediately after radiation, and the cell cycle was monitored for the following 72 h, by measuring cellular DNA content as published earlier (Pauwels *et al*, 2003c).

### Determination of apoptosis by Annexin V staining, *TUNEL* assay, and caspase 3 activity assay

Cells were plated in 75 cm^2^ culture flasks to ensure exponential growth during the experiments. After plating and a 24 h recovery period, ECV304 and H292 cells were treated with IC_25_ (2 and 4 nM, respectively) or IC_90_ (8 and 18 nM, respectively) concentrations gemcitabine for 24 h, immediately before or after radiotherapy (2 and 6 Gy). Seventy-two hours later, both adherent and detached cells were collected.

Annexin V staining was performed using Annexin V-FITC apoptosis detection kit I (Becton-Dickinson Pharmingen, Erembodegem, Belgium) in accordance with the manufacturer's instructions. Briefly, cells were washed twice with cold PBS, counted and 1 × 10^6^ cells were collected and resuspended in 1 ml binding buffer (10 mM HEPES/NaOH, 140 mM NaCl, 25 mM CaCl_2_). A measure of 100 *μ*l of this solution was mixed with 5 *μ*l Annexin V conjugated with fluorescein isothiocyanate (Annexin V-FITC) and 5 *μ*l propidiumiodide (PI). The cells were gently vortexed and incubated for 15 min at room temperature. Then, 400 *μ*l binding buffer was added. Analysis of green (Annexin V-FITC) and red (PI) fluorescence was performed in a FACScan flow cytometer (Becton-Dickinson).

To determine apoptosis by TUNEL assay, 2 × 10^6^ cells were collected and evaluated using *in situ* cell death detection kit, Fluorescein (Roche, Vilvoorde, Belgium) in accordance with the manufacturer's instructions. Briefly, cells were washed twice with PBS/1%BSA at 4°C. Then, 1 ml fixation solution was added to the cell suspension (1% formaldehyde) for 30 min on ice, while shaking. Cells were centrifuged (5 min, 1200 r.p.m.) and washed once with PBS. Cells were permeabilised in 70% ethanol for at least 30 min at −20°C until colouring. Cells were washed twice with PBS and incubated in TUNEL reaction mixture (dUTP-FITC) for 60 min at 37°C. Cells were washed again twice with PBS and resuspended in 500 *μ*l PBS containing 5 *μ*l PI/RNase (500 *μ*g ml^−1^ with 0.1% RNase). Samples were analysed in a FACScan flow cytometer, measuring green (dUTP-FITC incorporated in fragmented DNA) and red (PI binding to DNA) fluorescence of nuclei of individual cells.

Caspase 3 activity was determined using the colorimetric caspase 3 assay (Sigma Aldrich, Bornem, Belgium). This assay is based on the hydrolysis of the peptide substrate acetyl-Asp-Glu-Val-Asp p-nitroanilide (Ac-DEVD-pNA) by caspase 3, resulting in the release of pNA. pNA has a high absorbance at 405 nm (*ε*_nm_=10.5). The concentration of pNA released from the substrate is calculated from a calibration curve prepared with defined pNA solutions; 10^7^ control and treated cells were collected. Cells were washed with PBS and evaluated according to the manufacturer's instructions.

### Mitochondrial transmembrane potential measurement

The mitochondrial membrane potential (Δ*ψ*_m_) was flow cytometrically determined using a mitochondrial-sensitive probe: tetramethylrhodamine methylester (TMRM). It accumulates in the mitochondria and the transmembrane distribution of the dye is directly related to the membrane potential. Tetramethylrhodamine methylester does not accumulate in depolarised mitochondria. The extent of its uptake, measured as fluorescence intensity reflects Δ*ψ*_m_.

Cells were plated in 75 cm^2^ culture flasks as described above. Seventy-two hours after treatment with gemcitabine (IC_25_ and IC_90_) and/or radiotherapy (2 and 6 Gy), both adherent and detached cells were collected. After washing in PBS, cells were incubated with 200 nM TMRM and 5 *μ*l Annexin V-FITC for 15 min at 37°C and orange (TMRM) and green fluorescence were measured on a FACScan flow cytometer.

### Western blot and immunodetection

Cells were plated in 75 cm^2^ culture flasks to ensure exponential growth during the experiments. After plating and a 24 h recovery period, cells were treated with gemcitabine alone (IC_25_ and IC_90_), radiation alone (2–6 Gy), or the combination. At different time points after radiation, both adherent and detached cells were collected by trypsinisation; 10^6^ cells were washed with PBS and resuspended in 100 *μ*l laemmli sample buffer (Bio-Rad, Nazareth, Belgium). Western blotting and immunodetection were performed as described earlier (Pauwels *et al*, 2005b). Primary antibodies were 1/100 anti-caspase 8 (Ab-3) (Calbiochem, Leuven, Belgium), 1/100 anti-caspase 9 (Ab-2) (Calbiochem), 1/1000 anti-Bid and truncated Bid (tBid) (Bioké, Leiden, The Netherlands). Secondary antibodies were 1/1000 Anti-rabbit IgG peroxidase conjugate and 1/1000 anti-mouse IgG horse-radish-peroxidase linked (Bioké).

### Human apoptosis PCR array

A PCR array was used for gene expression analysis, taking advantage of real-time PCR performance, combined with the ability to detect the expression of many genes simultaneously.

Cells were plated in 75 cm^2^ culture flasks as described above. Seventy-two hours after treatment, total RNA samples were isolated from 4 × 10^6^ control and treated cells (IC_90_–6 Gy) or cells treated with an inducer of the extrinsic pathway (200 ng ml^−1^ TRAIL, 6 h, Calbiochem), using RNeasy Mini Kit (Qiagen, Venlo, The Netherlands). TRAIL-induced apoptosis was first confirmed after Annexin V staining (data not shown). The cDNAs were reversed transcribed from RNA using ReactionReady First Strand cDNA Synthesis Kit (SABiosciences, Tebu-Bio, Boechout, Belgium). Comparison of the relative expression of 84 apoptosis-related genes was characterised by human apoptosis PCR array (SABiosciences) and the RT^2^real-time SYBR Green/Rox PCR Master mix (SABiosciences) on a 7000 real-time PCR System (Applied Biosystems, Lennik, Belgium). The array includes the TNF ligands and their receptors, members of the Bcl-2 family, caspases, IAP, TRAF, CARD, DD, DED, and CIDE family, as well as genes involved in the p53 and ATM pathways (Supplementary Figure 1).

### Data analysis

Survival rates were calculated by mean OD (optical density) of treated cells/mean OD of control cells × 100%. The radiation survival curves were fitted according to the linear quadratic model: survival=exp(−*α*D–*β*D^2^), using Winnonlin (Pharsight, Mountain View, CA, USA). The radiosensitising effect was represented by the dose enhancement factor (DEF): ID_50_(−dFdC)/ID_50_(+dFdC). For the determination of synergism, the combination index (CI) was calculated by the Chou–Talalay equation (Pauwels *et al*, 2003a), using CalcuSyn (Biosoft, Cambridge, UK). A CI value between 0.9 and 1.1 indicates only additivity. Moderate synergism is depicted by CI values between 0.7 and 0.9, synergism by CI values below 0.7.

Flow cytometric data were analysed using Cell Quest (Becton-Dickinson) software.

Unless otherwise indicated, all data are presented as the mean±s.d. All experiments were performed at least three times. A two-sample *t*-test and two-way ANOVA were used to determine statistical significance (*P*<0.05). Two-way ANOVA was used to study the impact of the concentration of gemcitabine, doses radiotherapy, and treatment schedule on the outcome parameter (cell survival). *Post hoc* comparisons revealed which groups differed significantly from one another. A correlation coefficient was calculated between apoptosis induction and cell survival.

To analyse the data of the PCR array, a centroid-mediated classification algorithm was applied. Therefore, two centroids, representing the average gene expression pattern of the apoptosis-related genes with or without treatment with TRAIL, were calculated for H292 and ECV304 cells separately. Thereafter, the agreement between the apoptosis-related gene expression pattern of control cells or cells treated with gemcitabine and radiotherapy and the apoptosis-related gene expression pattern with or without treatment with TRAIL were quantified for each cell line. Therefore, Pearson correlation coefficients between the apoptosis-related expression pattern of each centroid (TRAIL-treated and TRAIL-untreated) and the apoptosis-related expression patterns of the cell lines (control or gemcitabine and radiation-treated cells) were calculated. Then, for each centroid separately, the correlation coefficients were compared between the control and the treatment conditions.

Next, *P*-values were calculated to identify individual genes with gene expression differences between the untreated and treated experiments for each cell line separately. Using the global test (Goeman *et al*, 2004), geneplots of the differentially expressed genes were generated to investigate the relative overexpression of each gene present in the gene list of differentially expressed genes. In addition, to account for the false discovery rate, the global test was used to calculate a *Z*-score for each of the differentially expressed genes between treated and untreated experiments. The *Z*-score represents the difference between the observed and expected (calculated by random class label permutations) gene expression differences between treated and untreated cells, normalised to the standard deviation of the distribution of expected gene expression differences.

## Results

### Cell survival after treatment with gemcitabine and radiation

**Figure 1:**
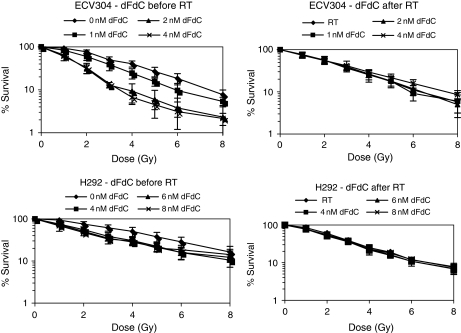
Radiation dose–response curves of ECV304 and H292 cells after treatment with gemcitabine (0–8 nM) during 24 h, immediately before or after radiotherapy (0–8 Gy).

A clear concentration-dependent radiosensitising effect of gemcitabine was observed in ECV304 and H292 cells, when cells were treated during 24 h with gemcitabine before radiotherapy (Figure 1). This radiosensitising effect was not observed when cells were treated with gemcitabine during 24 h immediately after irradiation. When gemcitabine treatment preceded radiation, DEFs ranged from 1.39 to 3.05 (CI values 1.05–0.65) in ECV304 cells and from 1.20 to 2.67 (CI values 1.21–0.76) in H292 cells (Pauwels *et al*, 2003a). When gemcitabine followed radiotherapy DEFs ranged from 0.98 to 1.02 and from 1.12 to 1.19 for ECV304 and H292 cells, respectively, with CI values above 0.827 for ECV304 and above 1.071 for H292 cells. Statistical analysis using two-way ANOVA revealed that cell survival was significantly influenced by the concentration of gemcitabine, the dose of radiation, and the treatment schedule (gemcitabine before or after radiotherapy) in both cell lines. *Post hoc* analysis showed in ECV304 significant differences among all radiation doses and also among 0, 1, and 2 nM gemcitabine. No significant difference was observed between 2 and 4 nM gemcitabine (*P*=0.865). In H292 cells, significant differences were observed among all radiation doses and between 0 and 4 nM gemcitabine. No significant differences were observed among 4, 6, and 8 nM gemcitabine (*P*>0.724).

### Cell cycle effect after the combination gemcitabine and radiation

**Figure 2:**
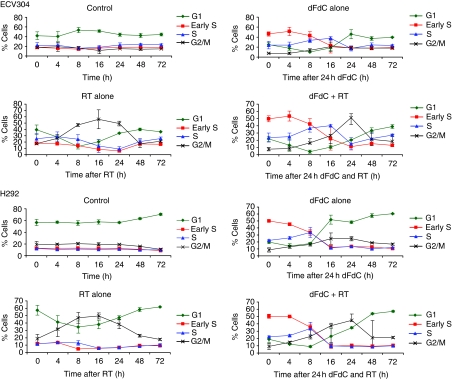
Cell cycle analysis at different time points after treatment with gemcitabine (8 nM), radiotherapy (4 Gy), or the combination of gemcitabine and radiotherapy.

In Figure 2, the distribution of ECV304 and H292 cells over the cell cycle phases at different time points is shown. Without treatment, the distribution of cells over the different phases is very similar over time. Treatment with gemcitabine (8 nM) alone resulted in an early S-phase block immediately after treatment, as we reported earlier (Pauwels *et al*, 2003c) and 8 h later, the amount of S-phase cells increased and 48 h after treatment the cell cycle distribution returned to pre-treatment levels. In ECV304 cells, radiation (4 Gy) caused a G_2_/M block, which was maximal 16 h after radiotherapy. In H292 cells, radiotherapy resulted in a G_2_/M block, which was also maximal 16 h after radiotherapy, whereas the amount of G_1_ cells remained roughly constant at the expense of S-phase cells. 48 h after radiation, the cell cycle distribution returned to pre-treatment levels. Treatment with the combination of gemcitabine and radiotherapy resulted in an early S-phase block immediately after treatment, both in ECV304 and in H292 cells. This block was followed by a synchronous movement of the cells to the G_2_/M phase. The G_2_/M block was maximal 24 h after treatment, being 8 h later than after radiotherapy alone. The cell cycle distribution returned to pre-treatment levels 72 h after combination treatment.

### Analysis of apoptosis induction after treatment with gemcitabine and/or radiotherapy

**Figure 3:**
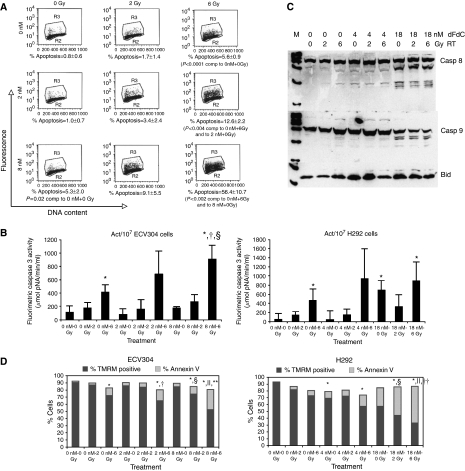
(**A**) TUNEL-positive ECV304 cells 72 h after treatment with gemcitabine, radiotherapy, or the combination of gemcitabine and radiotherapy. Dot plot from a representative experiment (R2=normal cell population, R3=apoptotic cell population). (**B**) Caspase 3 activity of ECV304 and H292 cells 72 h after treatment with gemcitabine, radiotherapy, or the combination of gemcitabine and radiotherapy. ^*^P<0.05 *vs* 0 nM–0 Gy, ^†^*P*<0.05 *vs* 0 nM–6 Gy, ^§^*P*<0.05 *vs* 8 nM–0 Gy. (**C**) Caspase 8, caspase 9, Bid and tBid expression of H292 cells 72 h after gemcitabine (IC_25_, IC_90_) and/or radiotherapy. Similar results were observed with ECV304 cells. (**D**) Annexin V-positive cells with reduced TMRM fluorescence (loss of Δ*ψ*_m_) and TMRM-positive cells after treatment with gemcitabine, radiotherapy, or the combination. ^*^P<0.05 *vs* 0 nM–0 Gy, ^†^*P*<0.05 *vs* 2 nM–0 Gy, ^§^*P*<0.05 *vs* 0 nM–2 Gy, ^∣∣^*P*<0.05 *vs* 0 nM–6 Gy, ^**^P<0.05 *vs* 8 nM–0 Gy, ^††^*P*<18 nM–0 Gy.

The amount of apoptotic cells was determined using Annexin V staining and caspase 3 activity assay. For ECV304 cells, apoptosis induction was also evaluated by TUNEL assay. In Table 1, the amount of Annexin V-positive cells is summarised. For both ECV304 and H292 cells, the amount of early apoptotic cells increased with the combination gemcitabine and radiation. More apoptotic cells were observed with a higher concentration gemcitabine or higher radiation dose. Similar results were observed with TUNEL assay for ECV304 cells (Figure 3A). In fact, treatment with 8 nM of gemcitabine and 6 Gy radiation resulted in more than 50% TUNEL-stained cells 72 h after treatment.

Using the caspase 3 activity assay, these results were confirmed. Caspase 3 activity increased with the combination of gemcitabine and radiotherapy (Figure 3B).

Although apoptosis was determined when cells were treated with gemcitabine immediately after radiation, Annexin V staining was less pronounced than when gemcitabine treatment preceded radiation (Table 1). In H292 cells, this is significantly different.

The amount of apoptosis of ECV304 and H292 cells was negatively correlated to the cell survival observed with the SRB test for the IC_25_ values of gemcitabine (the correlation coefficient was −0.8 and −0.9 for ECV304 and H292 cells, respectively, when apoptosis was determined with Annexin V staining). This means that there is a positive correlation between apoptosis induction and the amount of cell kill using lower concentrations of gemcitabine. The correlation coefficient for the IC_90_ values could not be calculated because this concentration was not used in the SRB test because of high toxicity.

### Immunodetection of caspase 8, caspase 9, Bid and tBid after treatment with gemcitabine and radiotherapy

To investigate the apoptotic pathway under radiosensitising conditions, the expression of caspase 8 and 9, initiator caspases of, respectively, the receptor and mitochondrial-mediated pathway were investigated at the moment apoptosis was observed, that is 72 h after combination treatment.

Gemcitabine alone induced the activation of caspase 8 in H292 and ECV304 cells. In H292, both IC_25_ and IC_90_ gemcitabine concentrations resulted in caspase 8 cleaving. No activation of caspase 9 could be shown after incubation with gemcitabine alone. A similar effect was observed after irradiation alone (data not shown). When cells were treated with the combination of gemcitabine and radiotherapy, cleaving products of both caspase 8 and 9 were observed. This could mean that both the extrinsic and intrinsic apoptotic pathway were involved in the apoptotic cell death. Therefore, Bid and tBid expression were determined. However, Bid expression was quite variable and did not show any relationship with treatment. No tBid could be shown (Figure 3C).

### Measurement of the transmembrane mitochondrial potential

In Figure 3D, TMRM-positive cells and Annexin-V-positive cells with a reduction in TMRM fluorescence (loss of Δ*ψ*_m_) are shown. Treatment with gemcitabine, radiotherapy but especially with the combination of both did increase the amount of cells with a loss of mitochondrial potential. Thus, induction of apoptosis under radiosensitising conditions is accompanied by decrease in Δ*ψ*_m_.

### Human apoptosis PCR array

**Figure 4:**
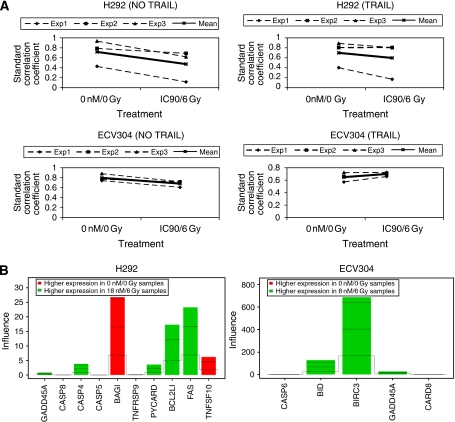
(**A**) Standard correlation coefficient of ΔΔCt values of ECV304 and H292 cells (0 nM–0 Gy and IC_90_-6 Gy), compared with the centroid of ECV304 and H292 cells (no TRAIL or TRAIL). Results of three different experiments and mean of the experiments are shown. (**B**) Global test for group of genes. Individual genes with significant gene expression differences between the untreated (0 nM–0 Gy) and treated (IC_90_-6 Gy) experiments are shown (*P*<0.05) for ECV304 and H292 cells.

In Figure 4A, the correlation coefficient of control and treated cells with the centroids (no TRAIL or TRAIL) are shown. When the correlation coefficient of treated cells (IC_90_-6 Gy) and the centroid without TRAIL are compared with the correlation coefficients of the treated cells (IC_90_-6 Gy) and the centroid with TRAIL, the latter correlation coefficients are significantly elevated. When the curve slopes were determined and compared for each cell line separately, there was a significant difference for ECV304 (*P*=0.002), whereas there was only a trend to difference for H292 cells (*P*=0.095). This means that the gene expression pattern of ECV304 after treatment with the combination of gemcitabine and irradiation was comparable with the gene expression pattern of cells treated with TRAIL. In H292 cells, gene expression pattern using radiosensitising conditions was also comparable with the pattern after treatment with TRAIL, however, to a lesser extent then in ECV304 cells.

In Figure 4B, the individual genes with significant gene expression differences between the untreated (0 nM–0 Gy) and treated (IC_90_-6 Gy) experiments are shown (*P*<0.05). In ECV304 cells, expression of caspase 6, BID, BIRC3, GADD45A, and CARD8 was more pronounced in samples of treated cells. In H292 cells, GADD45A, caspase 8, caspase 4, caspase 5, TNFRSF9, PYCARD, BCL2L1, and Fas showed a higher expression in cells after treatment with gemcitabine and radiotherapy. All these genes are pro-apoptotic genes, except BIRC3 for ECV304 cells and BCL2L1 for H292 cells.

## Discussion

In this study, the role of apoptosis in the radiosensitising effect of gemcitabine was investigated thoroughly. An increased induction of apoptosis was observed using radiosensitising conditions as a result of activation of the extrinsic or receptor-mediated apoptosis pathway. This is the first study investigating the apoptosis pathway after treatment of tumour cells with radiosensitising conditions of gemcitabine.

Apoptosis has shown to be a significant mode of cell death after tumour treatment and may play a significant role in drug and radiation enhancement (Tolis *et al*, 1999; Ostruszka and Shewach, 2000; Fukuoka *et al*, 2001; Lawrence *et al*, 2001; Liu *et al*, 2001). However, this might be the case only in some tumour types and might occur either as a primary event induced by therapy or as a secondary event after lethal damage to the cell (Brown and Wouters, 2001; Tannock and Lee, 2001) Increased induction of apoptosis is observed with the radiosensitising effect of taxanes (Creane *et al*, 1999), vinorelbine (Fukuoka *et al*, 2001; Zhang *et al*, 2004), campthotecin (Rich, 2003), and oxaliplatin (Hermann *et al*, 2008). However, clinical data do not provide definitive evidence for the role of apoptosis in the response to cancer therapy, and in fact contradicting reports were published, also concern the apoptotic pathway (Brown and Wilson, 2003). Although some studies assign a key role for the mitochondrial pathway in drug-induced apoptosis and the death-receptor pathway may amplify this, other studies suggest that mitochondria may act as amplifiers, but not initiators of cell death (Debatin and Krammer, 2004; Fulda and Debatin, 2006). However, further insight into the complex signalling network activated in response to anticancer therapy is necessary to see to what extent the current knowledge can be exploited for the design of new cancer therapies (Fulda and Debatin, 2006).

As showed earlier, a clear radiosensitising effect has been observed when gemcitabine treatment precedes irradiation (Shewach and Lawrence, 1996; Pauwels *et al*, 2005a). It has also been shown that the cell cycle effect of gemcitabine, being a block in the early S-phase, is correlated with the radiosensitising effect (Pauwels *et al*, 2005a). In this study the cell cycle progression after treatment with the combination of gemcitabine and radiotherapy initially resulted in an early S-phase block, after which the cells progressed through the cell cycle. Seventy-two hours after treatment, the cell cycle distribution returned to pre-treatment levels. We observed that the amount of cells in culture had decreased at that time and because the S-phase is not the most radiosensitive phase (Sinclair, 1972; Tang *et al*, 1997), we hypothesised that the blocked S-phase cells underwent apoptosis. An increased induction of apoptosis was observed using radiosensitising conditions as determined by Annexin V staining, caspase 3 activity assay, and TUNEL assay. When gemcitabine followed radiation, less apoptotic cells were observed. This schedule did not result in a radiosensitising effect either. It seemed that apoptosis was induced after completion of S and G2/M phase and that the cell cycle perturbations followed by the induction of apoptosis play an important role in the radiosensitising effect. Wachters *et al* have shown that gemcitabine causes radiosensitisation by specific interference with homologous recombination-mediated repair and that homologues recombination repair is preferentially used in late S and G2 phases of the cell cycle (Wachters *et al*, 2003), supporting our observations that apoptosis was induced after completion of these cell cycle phases. Gemcitabine is known to induce apoptosis by itself when used in cytotoxic concentrations (Huang and Plunkett, 1995b; Huang *et al*, 1997). However, additional involvement of apoptosis in the radiosensitising effect of gemcitabine has been reported also (Doyle *et al*, 2001; Lawrence *et al*, 2001; Zhang *et al*, 2004) and our findings are consistent with these reports. Lawrence *et al* concluded indeed that apoptosis plays an important role in gemcitabine-mediated radiosensitisation, but considered this not to be the sole mechanism responsible for radiosensitisation, based on the findings that the largest effect was observed in the apoptotic-prone HT-29 cells and only modest apoptosis in UMSCC-6 and A549 cells (Lawrence *et al*, 2001). Lawrence *et al* and Doyle *et al* showed that it is a misconception that a defect in apoptosis should restore a treatment-responsive state to the cell. They showed that cell death occurs according to the mode of death intrinsic to the cell line (Doyle *et al*, 2001; Lawrence *et al*, 2001). In our study, a correlation was found between the increase in apoptotic cells and the cell survival obtained with the SRB test with the IC_25_ concentrations of gemcitabine. The lesser response of H292 cells using the SRB test in Figure 1 in correlation with apoptosis induction may be explained by the different determination times; that is apoptosis was determined 72 h after treatment, whereas cell survival is determined after 7 days.

The study of Lawrence *et al* only investigated the terminal parts of the apoptotic pathway (caspase 3 activation and morphological changes). Apoptosis can results from the activation of several upstream pathways including TNF-receptor stimulation and mitochondrial release of cytochrome *c* (Green, 1998). The second part of our study was to determine which of these pathways were activated during radiosensitisation, because the possibility remained that selective potentiation of the active pathway could increase the effectiveness of gemcitabine as a radiation sensitiser (Lawrence *et al*, 2001).

Using radiosensitising conditions, both active caspase 8 and 9 could be observed by western blot. Therefore, the mitochondrial membrane potential was measured. These experiments confirmed the involvement of the mitochondria after treatment of the cells with the combination of gemcitabine and radiotherapy and because of these results, it seemed that gemcitabine and radiotherapy initially activated the extrinsic apoptosis pathway, but that amplification of this apoptosis signal by the mitochondrial pathway seemed required. Therefore, the activation of Bid protein was determined. Bid provides the only known connection between the extrinsic and the intrinsic pathways (Kuribayashi and El-Deiry, 2008). Unfortunately, the activated form tBid could not be observed. To further investigate in depth the pathway induced by radiosensitising conditions of gemcitabine, an apoptosis PCR array was performed. We could show that gene expression after treatment with gemcitabine and radiation was comparable with gene expression after treatment with TRAIL. This was significant in ECV304 cells. The similarity was less for H292 cells. TRAIL is a known inducer of the extrinsic apoptosis pathway (Thorburn, 2007). This could mean that in ECV304 cells (mt p53), the increased induction of apoptosis after the combination of gemcitabine and radiotherapy is a result of the activation of the extrinsic apoptosis pathway, whereas in H292 cells, which are wt p53 cells, this pathway is very likely activated, but another apoptosis pathway must be involved also. Tolis *et al* showed that gemcitabine exerts its antitumour effect by the apoptotic machinery both in wt and mt p53 cells, indicating the presence of p53 independent pathways (Tolis *et al*, 1999). The difference between the two cell lines was also observed when we looked to the genes that were significantly higher expressed in treated cells (Figure 4B). In H292 cells, GADD45A, caspase 8, and Fas were expressed more pronounced after treatment with gemcitabine and radiotherapy, and it is known that these genes are transcriptionally regulated by p53. GADD45A can also be induced by p53 independent mechanisms after DNA damage (source: http://www.ncbi.nlm.nih.gov/pubmed/, 2008) as observed in ECV304 cells. Using the PCR array, we observed an increase in expression of Bid in ECV304 cells after using radiosensitising conditions, whereas we could not observe this using a western blot. Most of the upregulated genes are involved in a caspase-dependent programmed cell death.

Studies investigating the mechanism of apoptosis induction after gemcitabine treatment alone have been published with variable outcome. Most studies showed the involvement of the death receptor pathway. Pace *et al* and Gazzaniga *et al* showed that gemcitabine controls tumour progression by an increased sensitivity of tumour cells to Fas-dependent apoptosis (Pace *et al*, 2000; Gazzaniga *et al*, 2007). However, Ferreira *et al* showed caspase 8 activation independently from Fas/fasL signalling (Ferreira *et al*, 2000b) and concluded that caspase 8 forms the apical and mitochondria-dependent step that subsequently activates downstream caspases (Ferreira *et al*, 2000a). In multiple myeloma cell lines, it was shown that gemcitabine induced apoptosis with caspase 3, 8, 9, and PARP cleavage, indicating that several mechanisms of action, including death receptor pathway and mitochondrial damage, are involved (Nabhan *et al*, 2002). In addition, in pancreatic cells, caspase 8 was activated before the breakdown of the mitochondrial transmembrane potential (Christgen *et al*, 2005). However, though these studies are in line with our observations, other studies found the involvement of other apoptotic signalling pathways (Chang *et al*, 2004; Habiro *et al*, 2004; Schniewind *et al*, 2004; Kurdow *et al*, 2005; Okamoto *et al*, 2007).

In summary, we could grant a role for the cell cycle perturbations and for the induction and mechanism of apoptosis in the radiosensitising effect of gemcitabine. It seems that the combination of gemcitabine and radiotherapy activates the extrinsic apoptosis pathway. The involvement of caspase 9, the intrinsic pathway, is a secondary event, possibly resulting in an enhancement of apoptosis. Apoptosis induction was very similar to the apoptotic pathway after TRAIL treatment. Because gemcitabine can enhance TRAIL-induced apoptosis (Seol *et al*, 2007), the use of gemcitabine with TRAIL combined with radiation could be promising. However, apoptosis does not represent the sole killing mechanism by which cancers are eradicated, and other modes of cell death, that is necrosis, autophagy, and mitotic catastrophe, are some forms of cell death that cannot be easily classified at present, and have to be considered also (Debatin and Krammer, 2004; Fulda and Debatin, 2006). The primary mechanisms behind gemcitabines radiosensitisation do not necessarily invoke the apoptotic machinery. Impairment of double strand break repair through the homologous recombination pathway (Wachters *et al*, 2003) or futile mismatch repair cycles at replication forks (because of precursor imbalance), or both may provoke apoptosis or result in mitotic catastrophe. Different forms of cell death should be considered when addressing the question of radiation-induced cell damage or cell survival after irradiation (Abend, 2003).

This study reveals important new insights into the mechanism of radiosensitisation, and can form a solid basis for further studies on the role of apoptosis signalling molecules in clinical samples using DNA or proteomic arrays. These studies are warranted to assess the impact of these molecular parameters on clinical outcome.

## Figures and Tables

**Table 1:** Percentage Annexin V-positive (early apoptotic) ECV304 and H292 cells 72 h after treatment with gemcitabine for 24 h and/or radiotherapy

**dFdC immediately before RT**	**0 Gy**	**2 Gy**	**6 Gy**
*ECV304*			
0 nM	9.17±2.38	10.86±1.74	18.76±5.87^*^
2 nM	10.20±1.36	11.18±2.33	17.19±2.30^†^
8 nM	14.28±1.68^*^	18.34±8.41^§^	31.83±7.31^§,∣∣^
*H292*			
0 nM	4.00±1.55	6.52±2.39	13.53±4.04^*^
4 nM	4.77±0.72	5.55±1.51	15.61±1.20^††^
18 nM	20.06±5.09^*^	26.94±5.34^**^	38.26±7.82^∣∣,§§^
			
*dFdC immediately after RT*			
*ECV304*			
0 nM	12.67±2.93	12.56±4.50	23.16±2.70
2 nM	5.73±3.40	13.06±4.57	19.18±7.33
8 nM	7.35±5.35	7.30±4.13	17.18±7.07
*H292*			
0 nM	4.02±1.94	5.68±4.37	9.49±4.66
4 nM	3.42±1.91	2.20±0.75	5.60±3.12
18 nM	5.26±1.24	7.24±5.55	11.42±1.33^§§^

^*^P<0.05 *vs* 0 nM+0 Gy, ^†^*P*<0.05 *vs* 2 nM+0 Gy, ^§^*P*<0.05 *vs* 8 nM+0 Gy, ^∣∣^*P*<0.05 *vs* 0 nM+6 Gy, ^**^P<0.05 *vs* 0 nM+2 Gy, ^††^*P*<0.05 *vs* 4 nM+0 Gy, ^§§^*P*<0.05 *vs* 18 nM+0 Gy.
