# Supplementary material for: The role of apoptotic cell death in the radiosensitising effect of gemcitabine
Source: Br J Cancer. 2009 Aug 11;101(4):628–36. doi: 10.1038/sj.bjc.6605145 (PMC2736812; doi:10.1038/sj.bjc.6605145)
Supplement: Supplementary Figure 1 [file 6605145x1.pdf]

Figure 1

ABL1	AKT1	APAF1	BAD	BAG1	BAG3	BAG4	BAK1	BAX	BCL10	BCL2	BCL2A1
BCL2L1	BCL2L10	BCL2L11	BCL2L2	BCLAF1	BFAR	BID	BIK	BIRC1	BIRC2	BIRC3	BIRC4
BIRC6	BIRC8	BNIP1	BNIP2	BNIP3	BNIP3L	BRAF	CARD4	CARD6	CARD8	CASP1	CASP10
CASP14	CASP2	CASP3	CASP4	CASP5	CASP6	CASP7	CASP8	CASP9	CD40	CD40LG	CFLAR
CIDEA	CIDEB	CRADD	DAPK1	DFFA	FADD	FAS	FASLG	GADD45A	HRK	IGF1R	LTA
LTBR	MCL1	NOL3	PYCARD	RIPK2	TNF	TNFRSF10A	TNFRSF10B	TNFRSF11B	TNFRSF1A	TNFRSF21	TNFRSF25
TNFRSF7	TNFRSF9	TNFSF10	TNFSF7	TNFSF8	TP53	TP53BP2	TP73	TRADD	TRAF2	TRAF3	TRAF4
B2M	HPRT1	RPL13A	GAPDH	ACTB	HGDC	RTC	RTC	RTC	PPC	PPC	PPC

Bea Pauwels
